# Rehearsal of tactile working memory: Premotor cortex recruits two dissociable neuronal content representations

**DOI:** 10.1002/hbm.25220

**Published:** 2020-10-03

**Authors:** Timo Torsten Schmidt, Pia Schröder, Pablo Reinhardt, Felix Blankenburg

**Affiliations:** ^1^ Neurocomputation and Neuroimaging Unit (NNU), Department of Education and Psychology Freie Universität Berlin Berlin Germany

**Keywords:** attentional refreshing, attentional refreshment, attention‐based refreshing, fMRI, frequency, inferior frontal gyrus, premotor cortex, prioritizing, rehearsal, somatosensory, superior parietal lobe, tactile, working memory

## Abstract

Recent working memory (WM) research has focused on identifying brain regions that retain different types of mental content. Only few neuroimaging studies have explored the mechanism of attention‐based refreshing, which is a type of rehearsal and is thought to implement the dynamic components of WM allowing for update of WM contents. Here, we took advantage of the distinct coding properties of the superior parietal lobe (SPL), which retains spatial layout information, and the right inferior frontal gyrus (IFG), which retains frequency information of vibrotactile stimuli during tactile WM. In an fMRI delayed match‐to‐sample task, participants had to internally rehearse sequences of spatial layouts or vibratory frequencies. Our results replicate the dissociation of SPL and IFG for the retention of layout and frequency information in terms of activation differences between conditions. Additionally, we found strong premotor cortex (PMC) activation during rehearsal of either stimulus type. To explore interactions between these regions we used dynamic causal modeling and found that activation within the network was best explained by a model that allows the PMC to drive activity in the SPL and IFG during rehearsal. This effect was content‐specific, meaning that the PMC showed stronger influence on the SPL during pattern rehearsal and stronger influence on the IFG during frequency rehearsal. In line with previously established PMC contributions to sequence processing, our results suggest that it acts as a content‐independent area that flexibly recruits content‐specific regions to bring a WM item into the focus of attention during the rehearsal of tactile stimulus sequences.

## Graphical abstract

In an fMRI delayed match‐to‐sample task, participants had to internally rehearse sequences of spatial layouts or vibratory frequencies of tactile stimuli. To explore interactions between task activated regions we used dynamic causal modeling and found that activation within the network was best explained by a model that allows the premotor cortex to drive activity in the SPL and IFG during rehearsal.

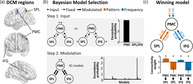

## 1 INTRODUCTION

The ability to flexibly represent and manipulate mental content is a key aspect of human cognition. It allows integrating information obtained from the senses with information from long‐term memory to solve complex cognitive tasks, perform reasoning, and make memory‐based decisions. Working memory (WM) studies contribute to an understanding of how the brain temporarily represents information.

A central question for current WM research is how higher cognitive functions can operate on WM content. On the one hand, answering this question requires understanding how WM content is coded as such. On the other hand, it requires identifying neuronal processes that relate to the update, manipulation, or change of mental content representations. An experimental approach to address this question capitalizes on a specific form of mental operation: mental rehearsal. Rehearsal is thought to be an important WM mechanism that allows maintaining WM content representations over extended delay periods. During rehearsal, participants retain multiple items and periodically pull individual items into the *focus of attention* (Oberauer, 2002), a process also referred to as *prioritizing of an item* (Myers, Stokes, & Nobre, 2017). Depending on the type of mental material that is rehearsed, this process can be distinguished into *articulatory rehearsal*, *elaborative rehearsal*, and *attention‐based refreshing* (Camos et al., 2018; Cowan, 1998; Oberauer, 2019). In *attention‐based refreshing*, which is addressed in the current study, nonverbal, sensory‐like mental material is retained, such as visual images or tactile sensations. According to the influential multicomponent model of WM (Baddeley & Hitch, 1974), such content is stored in *sensory buffer* systems. Neuronal activity that represents mental content in these buffer systems is thought to decay over time and *attention‐based refreshing* is thought to reactivate these neuronal ensembles and thereby act against the decay of delay activity and fading of a mental representation (Camos et al., 2018).

Multiple recent WM studies have challenged the idea of unitary buffer systems in the brain by testing which brain regions exhibit content‐specific activation during WM (Christophel, Klink, Spitzer, Roelfsema, & Haynes, 2017; Lee & Baker, 2016; Oberauer et al., 2018; Pasternak & Greenlee, 2005; Xu, 2017). While no direct mapping of particular brain regions to WM buffers has been established, it has been demonstrated that specific types of WM content, that is, specific stimulus attributes, can be decoded from anatomically distinct brain areas. In the tactile modality, we recently carried out two fMRI multivariate pattern analysis (MVPA) WM studies revealing that spatial layout and vibratory frequency information is retained in anatomically distinct brain regions. When participants memorized the spatial layout of vibrotactile stimuli, the superior parietal lobe (SPL) exhibited spatial layout specific codes (Schmidt & Blankenburg, 2018), whereas frequency information was coded in the right inferior frontal gyrus (IFG; Schmidt, Wu, & Blankenburg, 2017). Building on this clear‐cut anatomic dissociation now allows testing for interactions of these content‐coding brain regions with content‐independent regions that support the WM process independent of the maintained stimulus type.

In addition to brain regions that are activated in a content‐specific manner, multiple additional regions are well known to activate during WM irrespective of the particular types of retained content. However, the functional role of these regions is not well understood and they are mostly believed to serve general support functions, such as *cognitive control* or *attention allocation* (D'Esposito & Postle, 2015; Myers et al., 2017; Postle, 2015; Riggall & Postle, 2012). One potential mechanism of rehearsal could be that such areas reactivate content‐coding neuronal populations in regions processing particular stimulus attributes, either in the sense of attention‐based refreshing or by prioritizing one item out of multiple ones held in WM (Camos et al., 2018; Gazzaley & Nobre, 2012). A candidate region for this function is the premotor cortex (PMC) which is routinely found to be activated during WM (Carpenter, Baud‐Bovy, Georgopoulos, & Pellizzer, 2018; Marvel, Morgan, & Kronemer, 2019; Rottschy et al., 2012; Simon et al., 2002) and is involved in prospective attention during sequence processing (Schubotz, 2004). Recently, Fegen, Buchsbaum, and D'Esposito (2015) reported that activation in the PMC was modulated by WM load and rehearsal rate in a verbal WM study and we have repeatedly demonstrated the involvement of the PMC in tactile WM (Schmidt et al., 2017; Uluç, Velenosi, Schmidt, & Blankenburg, 2020; Velenosi, Wu, Schmidt, & Blankenburg, 2020; Wu et al., 2018). Together, these reports motivate further investigation into the functional contribution of the PMC to the rehearsal process, in particular its involvement in the reactivation of content‐specific codes.

The goal of the current study was to identify brain regions supporting WM storage and regions that support bringing a WM item into the focus of attention and probe their dynamic interaction during rehearsal. To identify relevant areas, we employed a delayed match‐to‐sample task in which participants rehearsed either vibratory frequency or spatial layout information while fMRI data was acquired. WM storage regions were expected to show content‐specificity, meaning that they activate specifically for their preferred stimulus modality, whereas regions supporting executive functions for bringing an item into the focus of attention were expected to activate independent of the rehearsed content. Based on our previous decoding studies we hypothesized that SPL and right IFG show content‐specific activation during rehearsal of spatial layout and vibrotactile frequency, respectively. Based on its ubiquitous activation in WM and previous rehearsal studies, we further hypothesized that the PMC activates during rehearsal, independent of the type of rehearsed stimulus information. Finally, we used dynamic causal modeling (DCM) to test whether mental continuation of vibrotactile stimulus sequences manifests in connectivity changes between content‐specific and content‐independent regions indicative of attentional recruitment of content‐coding neuronal ensembles during rehearsal.

## 2 MATERIALS AND METHODS

### 2.1 Participants

Seventeen healthy volunteers (mean age: 25.9 ± 6.4; 6 males, 11 females; 2 left‐handed) without any neurological or psychiatric disorder completed the study after giving written informed consent. The study adhered to the Human Subject Guidelines of the Declaration of Helsinki and was approved by the ethics committee of the Freie Universität Berlin. Participants were briefly familiarized with the task before participating in the study.

### 2.2 Experimental stimuli

**FIGURE 1:**
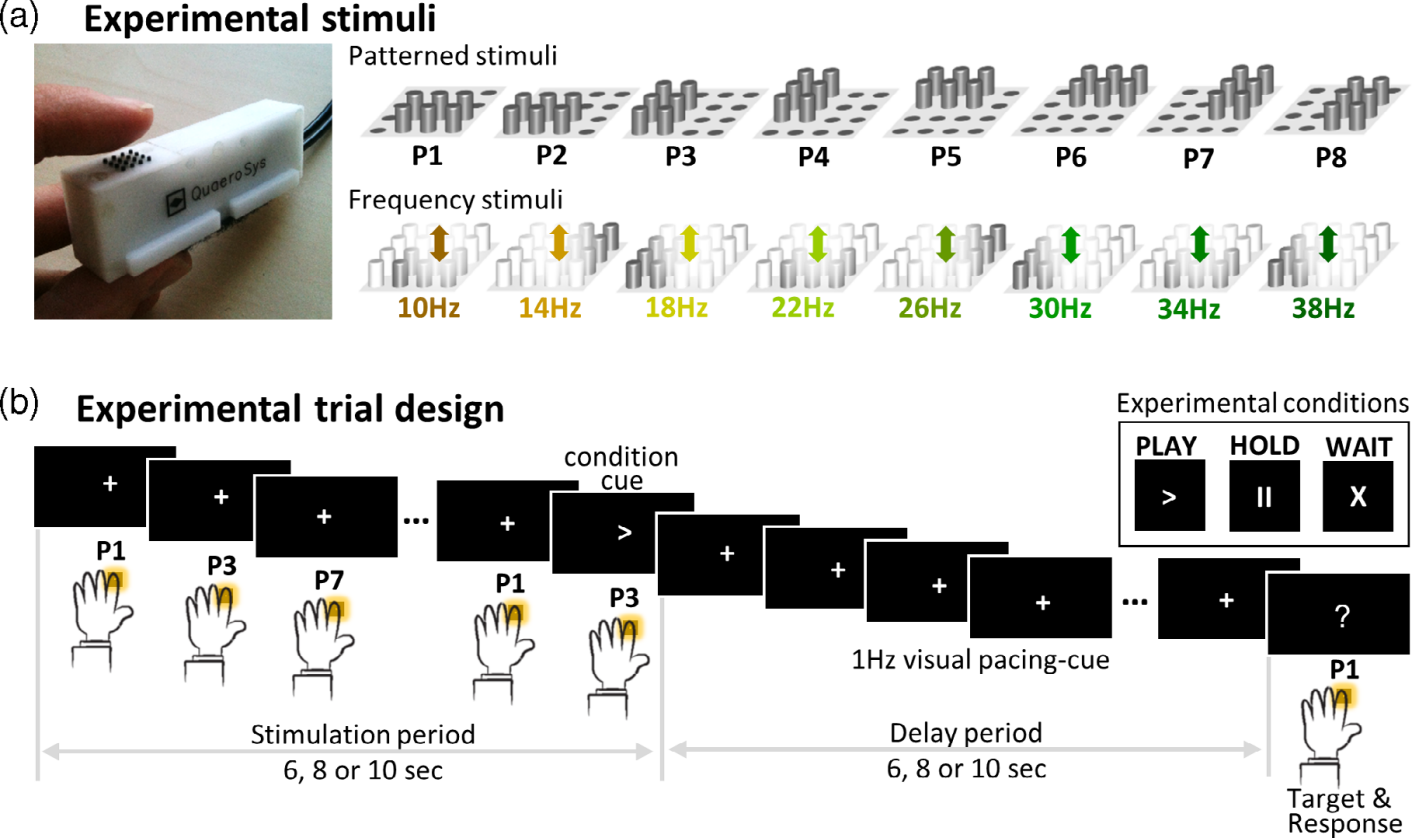
Experimental stimuli and trial design. (a) Vibrotactile stimuli were presented to the left index finger on a 4 × 4 pin Braille‐like display. Eight patterned and eight frequency stimuli were defined. For each trial, a stimulus sequence comprised three stimuli of either the pattern or the frequency stimulus set. (b) A 3 × 2 factorial design was employed with factors Condition (PLAY, HOLD, and CONTROL) and Stimulus type (PATTERN, FREQUENCY). Each trial started with a stimulation period where the trial‐specific stimulus sequence was repeatedly presented at 1 Hz pace. During this stimulation period, participants noticed if they were in a pattern or a frequency trial and encoded the stimulus sequence. A condition cue, presented with the last stimulus, indicated if participants had to mentally continue (rehearse) the sequence (PLAY), remember only the last stimulus (HOLD), or do nothing (CONTROL) until the presentation of a target stimulus. During the subsequent delay period, the fixation cross blinked at 1 Hz, serving as a visual guidance for the pace of rehearsal. Finally, participants indicated if a target stimulus was the currently rehearsed stimulus (PLAY), the maintained stimulus (HOLD), or a high/low‐frequency or upper/lower‐pattern stimulus (CONTROL) by making a button press. To perform these tasks, the PLAY condition required to actively rehearse the sequence of all three previously presented stimuli, in the HOLD condition only one stimulus was retained and the CONTROL condition did not require any memory, as the target stimulus decision task was not related to the stimuli presented during the stimulation phase

Vibrotactile stimulation was delivered using a 16‐dot piezoelectric Braille‐like display (4 × 4 matrix with 2.5 mm spacing) attached to the left index finger, and controlled by a programmable stimulator (Figure 1a, Piezostimulator, QuaeroSys, St. Johann, Germany). Stimuli were defined by eight different spatial patterns and eight stimulus frequencies. During stimulus presentation, a subset of the display pins vibrated for 600 ms with smoothened on‐ and offsets. For the pattern stimuli, the pins were driven at a frequency of 30 Hz and the patterns were defined by six vibrating pins as displayed in Figure 1a. Frequency stimuli also consisted of six pins, to match the amount of physical input, but the presented frequency varied across stimuli (Figure 1a). For each frequency‐trial, one stimulus pattern was randomly chosen from the given patterns and then all frequency stimuli of this trial were presented with the same spatial layout to exclude systematic influences of the spatial layout on the frequency‐trials.

### 2.3 Experimental paradigm

Participants performed a delayed match‐to‐sample task (Figure 1b) in which either a single vibrotactile stimulus had to be maintained or a sequence of stimuli had to be rehearsed. The study design constitutes a 3 × 2 factorial design with the factors *Condition* (PLAY, HOLD, CONTROL) and *Stimulus type* (PATTERN, FREQUENCY).

For each trial, a sequence of three stimuli was defined, either a sequence of frequency stimuli (FREQUENCY trials) or a sequence of patterned stimuli (PATTERN trials). To make the stimulus sequences more memorable and the stimuli sufficiently distinct, each sequence comprised two relatively similar stimuli and one more distinct stimulus. For the FREQUENCY sequences, we used two stimuli spaced by 8 Hz and the third stimulus by 16 Hz, resulting in the use of all permutations of order of the following sequences: [10 Hz 18 Hz 34 Hz], [14 Hz 22 Hz 38 Hz], [10 Hz 26 Hz 34 Hz], and [14 Hz 30 Hz 38 Hz]. For the PATTERN sequences we used two stimuli spatially overlapping in four pins and one stimulus that did not overlap with the other two (see Figure 1a), resulting in the use of all permutations of order of the following sequences: [P1 P2 P5], [P1 P2 P6], [P3 P4 P7], [P3 P4 P8], [P5 P6 P1], [P5 P6 P2], [P7 P8 P3], and [P5 P8 P4].

Each trial started with a stimulation period, during which the sequence was presented at a pace of one stimulus per second (1 Hz; interstimulus interval = 400 ms). Participants first had to notice if they were in a pattern or frequency trial based on the type of presented stimulus sequence. This difference was easy to notice as all stimuli in the PATTERN condition were presented at the same frequency, and all stimuli in the FREQUENCY condition with the same pattern. Importantly, they had to remember the presented stimulus sequence in the same way on every trial, as they did not know which of the three tasks they had to perform. The stimulation period lasted for 6, 8, or 10 s and ended with a visual condition cue indicating which task to perform. This cue was presented together with the last stimulus and indicated whether participants had to rehearse the sequence of stimuli (PLAY), retain only the last stimulus (HOLD), or wait without memorizing any information (CONTROL). The delay period lasted 6, 8, or 10 s. Throughout the delay period, the fixation cross changed its color between gray and white at 1 Hz to provide guidance for the pace of rehearsal. After the delay period, a target stimulus was presented. Depending on the task they had performed participants had to report if the target stimulus corresponded to the currently rehearsed stimulus in the PLAY condition or if the stimulus was identical to the single retained stimulus in the HOLD condition. As foil stimulus, one of the two nonmatching stimuli of the remembered sequence was presented as target stimulus. In the CONTROL condition, participants had to report if the stimulus was presented in the upper or lower half of the display in PATTERN trials (lower: P1, P2, P3, P8; higher: P4, P5, P6, P7); or if the frequency of the stimulus was high or low with regards to the range of frequencies in the stimulus set in FREQUENCY trials (lower: 10, 14, and 18 Hz; higher: 30, 34, and 38 Hz). Participants responded with a right index or middle finger button press, where the yes/no response‐mapping was randomized across participants. Participants were provided with visual feedback after every trial by shortly blinking “+” signs, meaning that the response was correct, or “−” signs, meaning that the response was incorrect, on both sides of the fixation cross. For both PLAY and both HOLD conditions, nine trials were presented per run, supplemented with three trials of each CONTROL condition, summing to 42 trials per run. Trial types were distributed randomly within each run. The inter‐trial‐interval varied between 2, 3, 4, 5, and 6 s.

The presentation of the condition‐cue at the end of the stimulation period ensured that participants had to process the stimulus sequence with the same attentional resources in all experimental conditions. The behavioral response allowed us to directly assess the successful rehearsal or retention and provided a behavioral measure for participants' commitment. Due to the variable length of the stimulation and delay periods, it was not possible for participants to predict the timing of the target stimulus.

### 2.4 fMRI data acquisition

Functional MRI data was acquired in three runs of 15 min 40 s on a 3T TIM Trio (Siemens) at the Center for Cognitive Neuroscience Berlin (CCNB). Four hundred and seventy functional volumes consisting of 37 slices were acquired per run using T2*‐weighted gradient‐echo EPI in interleaved order (TR = 2,000 ms; TE = 30 ms; 3 × 3 × 3 mm^3^ voxel; flip angle = 70°; 64 × 64 matrix). Additionally, a T1‐weighted MPRAGE with 176 sagittal slices, TR = 1,900 ms, TE = 2.52 ms, 1 × 1 × 1 mm^3^ voxel size was acquired.

### 2.5 General linear models

FMRI data were preprocessed with SPM12 (Wellcome Trust Centre for Neuroimaging, Institute for Neurology, University College London, UK). Functional data were slice time corrected, realigned to the mean image, normalized to MNI space using unified segmentation, interpolated to 2 × 2 × 2 mm^3^ voxel size, spatially smoothed with an 8 mm FWHM Gaussian kernel, and temporally detrended (Macey, Macey, Kumar, & Harper, 2004).

Statistical analysis was performed according to a standard general linear model (GLM) approach with SPM12. GLM regressors for the delay period of all trials of the FREQUENCY‐PLAY, FREQUENCY‐HOLD, FREQUENCY‐CONTROL, PATTERN‐PLAY, PATTERN‐HOLD, and PATTERN‐CONTROL conditions were included in the first‐level models. Further, we included regressors of no interest for the stimulation periods, responses, motion parameters and the first five principle components explaining white matter and cerebrospinal fluid signals, respectively. A control analysis was performed in which only trials with correct responses were modeled.

To test for pattern‐ and frequency‐specific activation, we computed first‐level contrasts for rehearsal (PATTERN‐PLAY versus FREQUENCY‐PLAY) and retention (PATTERN‐HOLD versus FREQUENCY‐HOLD) and forwarded the respective contrast images to second‐level one‐sample t‐tests to assess effects on the group level.

To test for activity related to the rehearsal process irrespective of the stimulus type, we computed a second‐level conjunction analysis. We used SPM's flexible‐factorial design option to specify a second‐level design including six first‐level baseline contrasts corresponding to the six task conditions and a subject factor. Within this model we specified the contrasts FREQUENCY‐PLAY > FREQUENCY‐HOLD and the PATTERN‐PLAY > PATTERN‐HOLD and computed a conjunction analysis of them testing against the conjunction null hypothesis (Friston, Penny, & Glaser, 2005). Similarly, to test for simple retention effects, we conjoined the FREQUENCY‐HOLD > FREQUENCY‐CONTROL and the PATTERN‐HOLD > PATTERN‐CONTROL contrasts. All activations are reported at *p* < .05, family wise error (FWE) corrected at the cluster level with a cluster‐defining threshold of *p* < .001. Thresholded statistical parametric maps were rendered on a standard 3D brain template using MRIcron (by Chris Rorden; Version 6 62,013). All reported coordinates correspond to MNI space. The SPM anatomy toolbox was used to establish cytoarchitectonical references where possible (Eickhoff et al., 2005; Eickhoff, Grefkes, Zilles, & Fink, 2007; Eickhoff, Heim, Zilles, & Amunts, 2006).

### 2.6 Dynamic causal modeling

The GLM analysis revealed a network of regions that activated during pattern and frequency rehearsal. To explain the emergence of this activity in terms of directed connectivity, we used DCM. DCM explains task evoked BOLD responses in a network of interconnected areas that is defined by direct driving inputs, fixed connectivity between areas, and modulation of this connectivity by experimental context. The activity in any given region is thus modeled as a weighted combination of its own previous state and inputs from other regions that may be stronger or weaker depending on experimental context. The network models are then endowed with a forward model mapping activity in the network to BOLD time courses in each region. Different model architectures (allowing for different input regions or connections) are then compared based on their aptitude to explain the observed BOLD time courses as quantified by their model evidence. The best model in the model space is identified by means of Bayesian Model Selection (BMS, Stephan, Penny, Daunizeau, Moran, & Friston, 2009) and its posterior parameter distributions can be used to infer the nature of connectivity changes induced by experimental conditions of interest.

Based on our GLM results and previous literature (Fegen et al., 2015; Schmidt et al., 2017; Schmidt & Blankenburg, 2018), we constructed models containing three regions: left PMC, left SPL, and right IFG. For each region and participant, activation time courses were extracted to be entered into the DCM analysis. To account for individual differences in the localization of activation peaks, the extraction of each participant's time course was adjusted to the individual's peak location within the activation cluster revealed by the group‐level analysis. We used corresponding routines of SPM's volume‐of‐interest extraction tools. Time courses from each region and each participant were extracted as follows: A group mask for the left SPL was defined by the PATTERN_PLAY > FREQUENCY_PLAY contrast, thresholded at *p* < .001 uncorrected and intersected with an anatomical SPL mask obtained from the Anatomy Toolbox to ensure anatomical specificity. Likewise, a group mask for the right IFG was defined by the FREQUENCY_PLAY > PATTERN_PLAY contrast, thresholded at *p* < .001 uncorrected and intersected with an anatomical IFG mask. Finally, a group mask for the left PMC was defined by the (FREQUENCY_PLAY > FREQUENCY_HOLD) and (PATTERN_PLAY > PATTERN_HOLD) conjunction contrast, thresholded at *p* < .001 uncorrected and intersected with an anatomical mask of the PMC obtained from Mayka, Corcos, Leurgans, & Vaillancourt (006). To extract one time course per participant and region, we reran first level GLMs on concatenated runs and determined individual peak voxels of the respective contrasts within the previously computed group masks. The first eigenvariate was then extracted from individually thresholded 4 mm radius spheres centered on these peaks (displayed in Figure 3a). This procedure ensured that time courses were extracted from voxels centered within group effect boundaries while still accounting for individual differences in exact peak locations.

Due to its content‐independent activation during rehearsal, we modeled the left PMC as a central node that is reciprocally connected to the content‐specific regions left SPL and right IFG (Figure 3a). To determine if pattern and frequency rehearsal had differential effects on the connectivity between these regions, we adopted a 2‐step procedure. In the first step, we tested whether the network was driven by input to content‐independent or content‐specific regions. Specifically, we constructed two models, one which allowed both pattern and frequency rehearsal to drive left PMC and one which assumed that pattern rehearsal would drive left SPL and frequency rehearsal would drive right IFG (Figure 3b, top panel), while keeping the connections between regions fixed. We then compared these two models by means of random effects BMS (Stephan et al., 2009). The winning model as indexed by model exceedance probabilities (EPs) then determined the input regions in the subsequent analysis. In the second step, we addressed potential connectivity modulation by experimental context. To this end, we allowed reciprocal PMC‐SPL and PMC‐IFG connections to take one of three states: fixed, modulated by pattern rehearsal, or modulated by frequency rehearsal (Figure 3b, bottom panel). With four connections and three possible states, this resulted in 3^4^ = 81 models that were compared using random effects BMS. The resulting EPs are reported for all models (Figure 3b). Connectivity weights of the winning model were extracted and tested for significance across participants using one‐sample t‐tests (corrected for false discovery rate [FDR, Benjamini & Hochberg, 1995] at FDR < 0.05). To address potential model dilution due to the large model space and ensure robustness of the results, we further assessed the effects of pattern and frequency rehearsal on each connection by performing four iterations of family‐level BMS (Penny et al., 2010), one for each connection. On each iteration, models were grouped based on their modulation of the respective connection (fixed, pattern, frequency; resulting in 27 models per family) and family EPs are reported.

## 3 RESULTS

### 3.1 Performance

Participants' performance in the six task conditions, expressed as percentage of correct responses, was as follows: FREQUENCY‐PLAY: 74.3 ± 13.5% (mean ± SD), PATTERN‐PLAY: 71.9 ± 13.6%, FREQUENCY‐HOLD: 78.4 ± 7.6%, PATTERN‐HOLD: 81.3 ± 8.8%, FREQUENCY‐CONTROL: 79.1 ± 18.0%, PATTERN‐CONTROL: 62.1 ± 21.2%. A 2 × 3 repeated measures ANOVA with Greenhouse–Geisser correction revealed a main effect of task condition (*F*[1.62, 25.94] = 5.79, *p* = .012), and posthoc *t*‐tests revealed the performance in the PATTERN‐CONTROL condition to be significantly lower (*p* < .01) than the PATTERN‐HOLD and FREQUENCY‐HOLD conditions (note however, that the control condition comprised only three trials per run, that is, nine trials in total, and was thus associated with high variance). Inspection of individual performance scores revealed that the effect was driven by only four participants who showed below chance‐level performance in the PATTERN‐CONTROL condition but normal performance levels in the other conditions. To ensure that these data sets did not bias our fMRI analysis we performed a control analysis, which included only trials with correct responses.

### 3.2 General linear models

**FIGURE 2:**
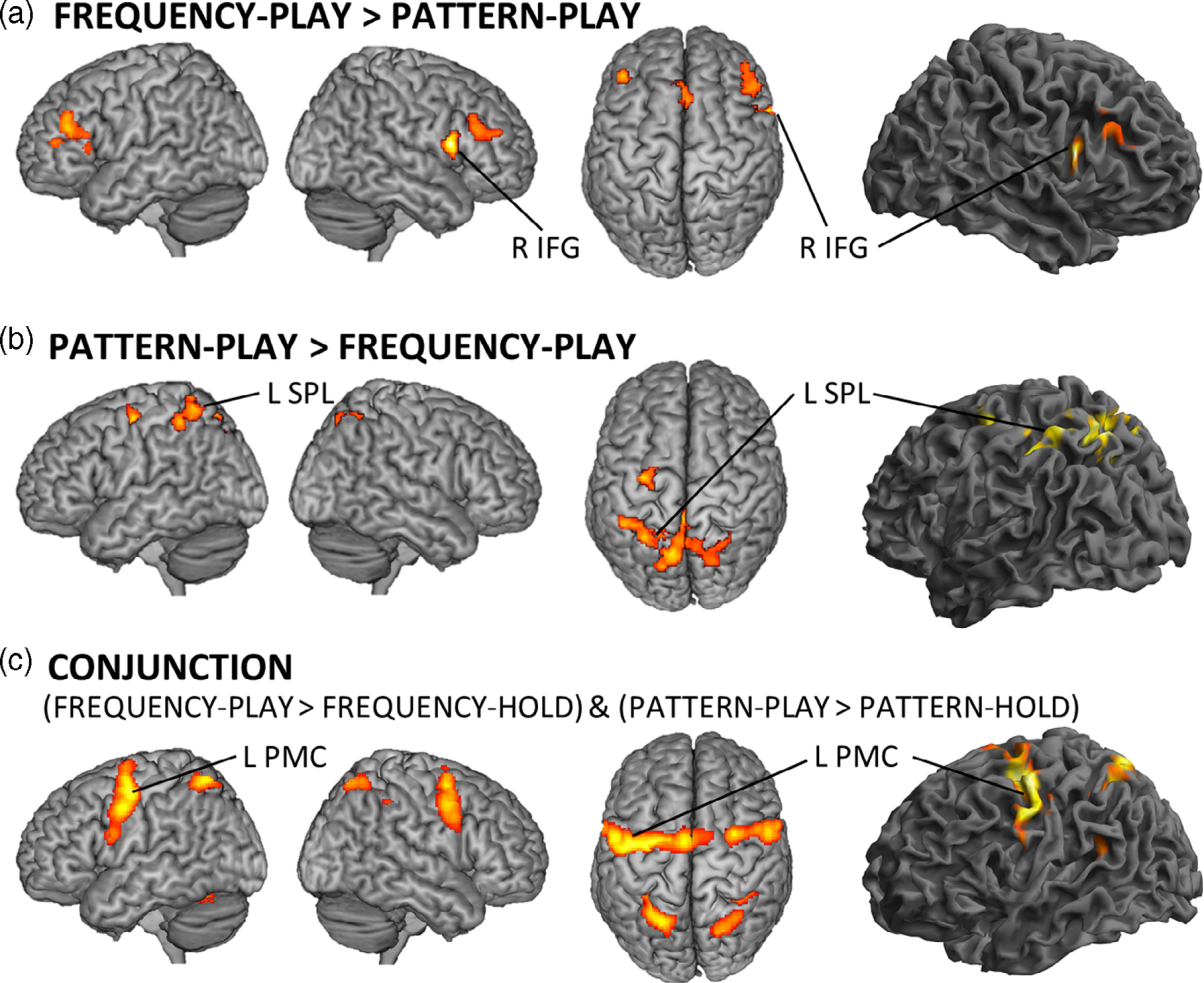
Brain activation during rehearsal of working memory representations. (a) The right inferior frontal gyrus (IFG) exhibits higher activity during frequency rehearsal than during pattern rehearsal. (b) The left superior parietal lobe (SPL) exhibits higher activity during pattern rehearsal than during frequency rehearsal. (c) The conjunction analysis tests for rehearsal activity irrespective of the specific rehearsal content. The strongest effect was found in the left premotor cortex (PMC). All clusters are presented at *p* < .05, family wise error (FWE) corrected at the cluster level with a cluster‐defining threshold of *p* < .001

To test for differences between the rehearsal of pattern stimuli and the rehearsal of frequency stimuli, we computed contrasts of the respective rehearsal periods. In line with our hypothesis, the rehearsal of frequency information activated the right IFG, while the rehearsal of pattern information activated the left SPL (Figure 2a,b, Table 1). Likewise, contrasting simple retention (HOLD condition) of pattern stimuli and frequency stimuli, revealed significantly stronger activation in the left SPL when spatial layout information was remembered (cluster size: 197 voxel; peak: *x* = −10; *y* = −56; *z* = 58) and significantly stronger activation in the right IFG when frequency information was remembered (cluster size: 208 voxel; peak: *x* = 50; *y* = 36; *z* = 26).

**TABLE 1:** Brain activation during rehearsal of WM representations

		Peak			
Cluster size	Region	MNI (*x*,*y*,*z*)			*t*
*PATTERN‐PLAY > FREQUENCY‐PLAY*					
932	L SPL area 7A	−10	−62	52	6.24
	L Precuneus	−2	−40	48	5.60
435	L SPL area 7PC	−28	−48	46	5.61
	L SPL area 7PC	−30	−52	66	5.40
212	L Precentral gyrus	−30	−10	56	5.77
105		34	−36	30	6.08
*FREQUENCY‐PLAY > PATTERN‐PLAY*					
393	L MFG	−42	36	22	5.45
257	L SMG	−8	30	44	7.05
226	R IFG (BA 45)	50	32	24	4.68
	R MFG	44	28	32	4.50
218	R IFG (BA 44)	54	12	14	8.05
*CONJUNCTION (FREQUENCY‐PLAY > FREQUENCY‐HOLD) and (PATTERN‐PLAY > PATTERN‐HOLD)*					
2,337	L PMC	−28	−6	54	7.01
799	R PMC	30	0	58	5.89
602	L SPL	−24	−60	62	6.11
227	R SPL	22	−68	60	4.99
183	L Cerebellum	−26	−60	−26	5.36
149	R IPS	34	−44	46	5.19

*Note:* Activated clusters as displayed in Figure 2. All results are reported at *p* < .05 FWE‐corrected at the cluster level with a cluster defining threshold of *p* < .001.

Abbreviations: BA, Brodman area; IFG, inferior frontal gyrus; IPL, inferior parietal lobule; IPS, inferior parietal sulcus; MCC, middle cingulate cortex; MFG, medial frontal gyrus; PMC, premotor cortex; SMG, superior medial gyrus; SPL, superior parietal lobule.

To test for regions that are activated during rehearsal independent of the specific type of rehearsed information, we computed a conjunction of the contrasts comparing rehearsal to retention conditions: (FREQUENCY‐PLAY > FREQUENCY‐HOLD) and (PATTERN‐PLAY > PATTERN‐HOLD). Activated regions included bilateral PMC, bilateral SPL, left cerebellum, and right intraparietal sulcus (IPS), with the left PMC being the largest cluster (Figure 2c, Table 1). To test if the retention of a single WM item similarly activates the PMC, we computed the (FREQUENCY‐HOLD > FREQUENCY‐CONTROL) and (PATTERN‐HOLD > PATTERN‐CONTROL) conjunction. This conjunction analysis did not reveal PMC activation when investigated at *p* < .05 FWE corrected at cluster level, nor at an uncorrected level of *p* < .001.

The control analysis, modeling only trials with correct responses, confirmed our main findings by revealing virtually identical results, in particular highly similar clusters in R IFG (BA 44) (cluster size: 216, peak: [46 30 24], *t*‐score: 6.16); L SPL (cluster size: 437, peak: [−32 –52 60], *t*‐score: 5.22); L PMC (cluster size: 2057, peak: [−18 –6 56], *t*‐score: 6.62) in the respective contrasts of Figure 2c, Table 1.

### 3.3 Dynamic causal modeling

**FIGURE 3:**
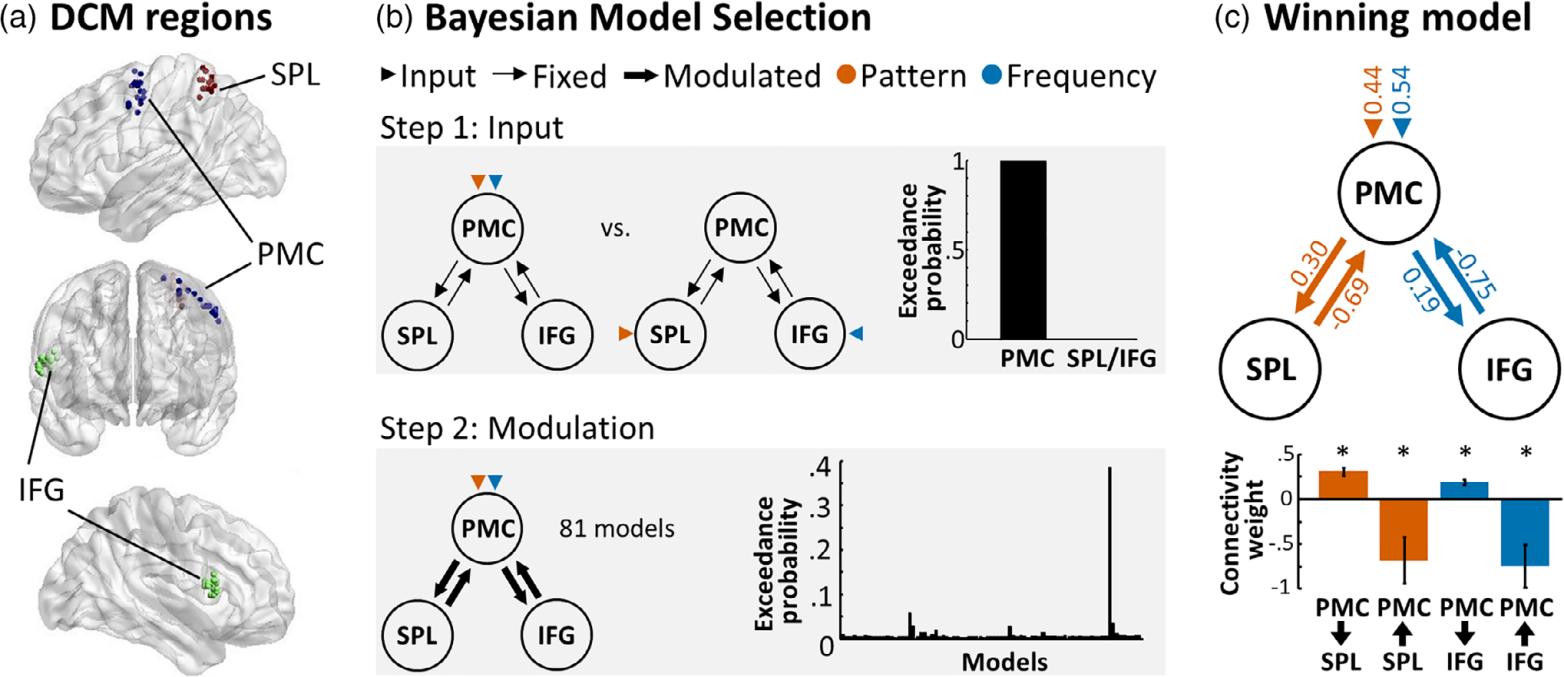
Dynamic causal modeling (DCM) of pattern and frequency rehearsal. (a) The regions included in the DCMs were defined based on the general linear model (GLM) analysis combined with region specific anatomical masks. Activation peaks for each participant in the premotor cortex (PMC), left superior parietal lobe (SPL) and right inferior frontal gyrus (IFG) are displayed. (b) Step 1: two input models were compared, allowing for driving input to either PMC or SPL/IFG. Random effects Bayesian Model Selection identified the left PMC as the most likely input region. Step 2: effects of pattern and frequency rehearsal on connectivity between regions were addressed in 81 DCMs that differed in their specific connectivity modulations (fixed, pattern, or frequency). The DCM allowing for modulation of recurrent PMC‐SPL connections by pattern rehearsal and recurrent PMC‐IFG connections by frequency rehearsal outperformed all other models (EP = 38.55%). (c) Parameter estimates of the winning model show positive connectivity modulations from PMC to SPL and from PMC to IFG, suggesting excitation, and negative connectivity modulations from SPL to PMC, suggesting inhibition. Error bars represent SEMs. Asterisks mark significant deviation from zero (*p* < .05 FDR‐corrected across 10 tests, FDR‐threshold: *p* = .026)

The GLM analysis revealed that the left PMC was activated during stimulus rehearsal independent of stimulus type, whereas the left SPL activated specifically during pattern rehearsal and the right IFG activated specifically during frequency rehearsal. Due to previous evidence for a role of the PMC in tactile WM and the rehearsal process, we focused our connectivity analyses on the interaction of the PMC with content‐specific regions. Comparing the two input models revealed that the model allowing for direct input into PMC clearly outperformed the alternative model assuming stimulus‐specific input to SPL/IFG (EP_PMC_ = 99.93%, EP_SPL/IFG_ = 0.07%; Figure 3b, top panel). All further models were therefore constructed with pattern and frequency rehearsal directly driving PMC. To test for connectivity changes in the network that can be indicative of the functional hierarchical organization of the rehearsal process, we compared 81 DCMs, each representing a different effect of experimental conditions on the connectivity between PMC‐SPL and PMC‐IFG. Random effects BMS resulted in the highest EP for the model allowing for modulation of the PMC‐SPL connections by pattern rehearsal and modulation of the PMC‐IFG connections by frequency rehearsal (EP = 38.55%; compare EP of the second best model: EP = 5.76%; Figure 3b, bottom panel). Thus, the winning model clearly outperformed all 80 other models in comparison. To ensure that these results were robust across the model space, we performed family‐level BMS on each connection. Consistent with the previously identified model, the PMC‐SPL connections were best explained by the model family allowing for a modulation by pattern rehearsal (PMC → SPL: EP_fixed_ = 0.36%, EP_pattern_ = 94.25%, EP_frequency_ = 5.39%; PMC ← SPL: EP_fixed_ = 0.18%, EP_pattern_ = 99.14%, EP_frequency_ = 0.68%), whereas the PMC‐IFG connections were best explained by the model family allowing for a modulation by frequency rehearsal (PMC → IFG: EP_fixed_ = 0.61%, EP_pattern_ = 1.24%, EP_frequency_ = 98.15%; PMC ← IFG: EP_fixed_ = 3.47%, EP_pattern_ = 0.14%, EP_frequency_ = 96.39%). To assess the specific effect of rehearsal on these connections we extracted connectivity weights from the winning model (Figure 3c). As expected, frequency and pattern rehearsal showed direct driving effects on PMC (frequency input: *w* = 0.44 ± 0.26, *t*(16) = 7.14, *p* < .001; pattern input: *w* = 0.54 ± 0.37, *t*(16) = 6.15, p < .001). Connections from the PMC to content‐specific regions showed significant positive modulation by rehearsal (PMC → SPL: *w* = 0.30 ± 0.16, *t*(16) = 7.81, *p* < .001; PMC → IFG: *w* = 0.19 ± 0.12, *t*(16) = 6.33, *p* < .001) whereas connections from content‐specific regions to the PMC showed significant negative modulation (PMC ← SPL: *w* = −0.69 ± 1.06, *t*(16) = −2.67, *p* = .017; PMC ← IFG: *w* = −0.75 ± 1.00, *t*(16) = −3.11, *p* = .007). We observed a similar but less pronounced pattern of connectivity weights in the DCM's A‐matrix, which models baseline connectivity irrespective of experimental conditions (PMC → SPL: *w* = 0.19 ± 0.23, *t*(16) = 3.53, *p* = .003; PMC ← SPL: *w* = −0.41 ± 0.33, *t*(16) = −5.11, *p* < .001; PMC → IFG: *w* = 0.01 ± 0.11, *t*(16) = 0.22, *p* = .83 (n.s.); PMC ← IFG: *w* = −0.13 ± 0.21, *t*(16) = −2.45, *p* = .026). This pattern suggests that the observed interactions may be a general property of the network, which is amplified during rehearsal (note that experimental and nuisance variables not included in the DCMs are regressed out, making it unlikely that unmodeled task components such as the HOLD conditions would have caused this effect).

## 4 DISCUSSION

In the current study, we investigated the neural underpinnings of tactile rehearsal. We replicate previous findings suggesting that the SPL processes spatial layout information whereas the right IFG processes frequency information during WM and extend this distinction to conditions of active rehearsal of tactile stimulus sequences (Figure 2a,b). In line with our hypothesis, we found the PMC to show strong activity during rehearsal, irrespective of the type of rehearsed content (Figure 2c). Within the rehearsal network, DCM identified the PMC as the most likely input region, which drives activity in SPL and IFG in a content‐specific manner. Taken together, our results suggest that the rehearsal process manifests in the interplay between the content‐independent region PMC with the content‐specific regions SPL and IFG.

Models of WM suggest the distinction between the representation of WM content (also termed WM storage) and dynamic components of WM for the update or manipulation of content. For example, the influential *Multicomponent Model of WM* suggests a distinction between buffer systems (e.g., the *visuospatial sketchpad*, *episodic buffer*, and *phonological loop*) and the *central executive*, which orchestrates WM operations on these buffer systems and interactions with other brain functions (Baddeley, 2012). A distinction between dynamic components and content codes can also be found in Cowan's *Embedded Processing Model of WM*. This model presumes that *attentional mechanisms* operate on long‐term memory traces or perceptual processes and reactivate these to bring a WM content into the focus of attention (Cowan, 2009; Ma, Husain, & Bays, 2014). Finally, attentional mechanisms that act on content representations are also presumed in models of mental imagery (Kosslyn, 2005), where attentional mechanisms are proposed to construct or initiate a mental image, which is then maintained as the content of WM (Tong, 2013). Taken together, the conceptual distinction between attentional mechanisms that implement dynamic components of WM and activity in neuronal ensembles that code specific WM content is a common assumption of WM models. The rehearsal process studied here amplifies the dynamic component of WM by continuously updating the specific mental content that is in the focus of attention at any given time. By employing different types of mental content that are well known to be represented in different brain regions (tactile frequency vs. spatial layout information), we capitalized on this process to experimentally dissociate brain regions supporting dynamic aspects of WM from those showing content representations. In line with this distinction, we identified the left PMC to activate independently of the rehearsed content and the right IFG and left SPL to activate in a content‐specific manner. We interpret this dissociation as reflecting dynamic components of WM and content storage, respectively.

### 4.1 Content‐specific activation in SPL and IFG

A solid body of human fMRI MVPA studies exists to support the observation that different brain regions code different types of material retained in WM (reviews: Lee & Baker, 2016; Christophel et al., 2017). Our results further support the notion that this also holds for tactile WM, where neuronal ensembles in different brain regions retain different stimulus properties of vibrotactile stimuli. The retention of vibratory frequency has been studied exhaustively with diverse methodology in nonhuman primates (for an overview, see Romo & de Lafuente, 2012) as well as in humans (e.g., Spitzer, Wacker, & Blankenburg, 2010). These studies have demonstrated that neuronal assemblies in the right IFG exhibit activity specific to the vibratory frequency retained in WM. Romo, Brody, Hernández, and Lemus (1999) found neurons with frequency‐modulated parametric firing behavior in intracranial recordings in nonhuman primates. Later, Spitzer et al. (2010) showed frequency modulated parametric beta band oscillations in a human EEG study and von Lautz et al. (2017) in a human MEG study. Likewise, human fMRI MVPA studies have revealed and replicated frequency‐specific codes in the right IFG (Schmidt et al., 2017; Wu et al., 2018). Finally, a TMS study demonstrated interference with WM performance after right IFG stimulation (Auksztulewicz, Spitzer, Goltz, & Blankenburg, 2011). One previous vibrotactile fMRI study by Spitzer, Goltz, Wacker, Auksztulewicz, and Blankenburg (2014) tested for IFG activation in a task where the WM content had to be actively changed over time. During a delay phase, participants had to mentally continue an increasing/decreasing amplitude modulation of vibratory stimuli. As during rehearsal, this mental continuation requires activating different content coding neuronal ensembles over time, and in accordance with previous WM studies, the authors observed delay activity in the IFG. In summary, the retention of vibrotactile frequency is a well‐characterized WM task with a consistent demonstration of content‐specific codes in the IFG.

Similarly, the retention of spatial stimulus features has previously been explored in the tactile modality. Activation in SPL/IPS was found during perception and mental imagery of spatial stimulus features (Schmidt & Blankenburg, 2019; Schmidt, Ostwald, & Blankenburg, 2014). In a recent MVPA study, we demonstrated that spatial layout information can be decoded from activation patterns in the SPL when participants memorized vibrotactile stimuli similar to those used in the current study (Schmidt & Blankenburg, 2018). These findings complement reports from visual WM, where representations of spatial stimulus properties were observed in posterior parietal regions (Christophel, Cichy, Hebart, & Haynes, 2015; Christophel, Hebart, & Haynes, 2012), and align well with the presumed functional role of the SPL/IPS for processing and remapping egocentric and allocentric spatial coordinate systems in nonhuman primates and humans (Grefkes & Fink, 2005; Heed, Buchholz, Engel, & Röder, 2015).

Taken together, our data and previous literature suggest that activation in the rIFG and SPL relate so the representation of WM content. However, this does not imply that these regions necessarily act as *buffers* or represent mental content independent of other regions. Instead, it is likely that a mental content is represented as joint activation of interacting neuronal ensembles across multiple regions. This view is supported by our analysis, which did not only reveal rIFG and lSPL but included additional prefrontal and posterior clusters. Also, previous studies have indicated additional regions to be involved in tactile WM representations, for example, the primary somatosensory cortex (Katus & Eimer, 2015; Katus, Grubert, & Eimer, 2015; Tamè & Holmes, 2016) at least during early phases of WM retention (Schmidt & Blankenburg, 2018). Here, we focused our further analysis on the regions with the strongest activation and best literature support to explore basic principles of rehearsal related network interactions. Future research is necessary to test what roles the additionally involved regions serve with regards to content representation or the rehearsal process.

### 4.2 Content‐independent activation in the PMC

With the introduction of stimuli conveying both vibrotactile frequency and spatial layout information, our study allows distinguishing between content‐specific and content‐independent activity. To assess content‐independent activity, we computed the conjunction analysis of PLAY > HOLD conditions (Figure 2c). The identified network strongly overlaps with the so‐called task‐positive network, a set of brain regions that activate during many cognitive neuroscience tasks and that have been functionally associated with domain‐general, attentional contributions (Fox et al., 2005). The strongest activation cluster in this analysis was found in the left PMC with some extensions into the caudal prefrontal cortex. The core of the identified cluster matches well with central and ventral aspects of the dorsal PMC as delineated in a multimodal parcellation study (Genon et al., 2018). While the PMC activation cluster connects to a cluster in the SMA, it does not include the eye movement‐related rostro‐ventral aspects of the PMC depicted in (Genon et al., 2017; Genon et al., 2018), and did not overlap with the inferior frontal junction, as delineated in Muhle‐Karbe et al. (2016). Similar activation clusters in the PMC are commonly found across different WM tasks, where different studies present distributed activation across the dorsal PMC (for a review see Marvel et al., 2019, for a meta‐analysis see Rottschy et al., 2012). The distribution of PMC activation peaks across participants in our study can be seen in Figure 3a as depicted for the DCM analysis.

While previous research has primarily focused on the importance of the PMC for the preparation and execution of movements (e.g., temporal aspects of motor sequences), it has also been found to be involved in nonmotor processes such as WM and spatial attention (for a review see Simon et al., 2002). Importantly, the PMC has previously been reported to support sub‐vocal rehearsal processes (Fegen et al., 2015). Interestingly, Schubotz (2004) have described the PMC as a core region allowing for prospective attention in sequential processing of different modalities. In their task, participants had to detect violations in repeatedly presented sequences of stimuli, which requires prospective attention in a similar fashion as is required for rehearsal. In these studies, PMC activity was not related to the task difficulty but rather to the processing of sequence information as such (Schubotz & Von Cramon, 2002). With regards to mental imagery, two fMRI studies in the tactile modality have revealed PMC involvement when stimulation at different body locations (Schmidt & Blankenburg, 2019) or stimuli with detailed spatial patterns on the left index finger (Schmidt et al., 2014) were imagined.

Due to the nature of the task, our rehearsal (PLAY) and WM (HOLD) conditions had different WM loads (three items had to be retained in the PLAY condition but only one item in the HOLD condition). Therefore, the GLM analyses cannot unequivocally assign the activity differences in the PMC to rehearsal processes. A previous rehearsal study by Fegen et al. (2015) indeed found PMC activity to be modulated by WM load, however mainly in an early phase of a long rehearsal period and together with a broader frontal network of regions. Importantly, PMC activity in that study was also modulated by the rehearsal rate and this modulation was stable throughout the long rehearsal period, suggesting that the activity observed in PMC was at least in part driven by rehearsal processes. Likewise, in studies on sequence processing, PMC activity was not found to be related to load but rather to the processing of sequence information as such (Schubotz & Von Cramon, 2002). In our study, the crucial difference between the PLAY and HOLD conditions was that participants had to shift their focus of attention between different WM items only in the PLAY condition but not in the HOLD condition where the same item had to be maintained throughout the delay period. This means that in the PLAY condition different neuronal populations coding different WM items had to be alternatingly reactivated, whereas in the HOLD condition the same neuronal population had to be active throughout the delay (note however that even in the HOLD condition participants may have occasionally switched their attentional focus between the item to be maintained and task‐irrelevant distractors, but this effect was expected to be considerably smaller than in the PLAY condition, where a repeated and continuous switch of attentional focus between items was necessary to successfully complete the task). Thus, the PLAY and HOLD conditions did not only differ in their WM load but in the dynamic reactivation of neuronal ensembles and this difference is expected to manifest in activation differences in brain regions implementing such dynamic processes. Moreover, we observed no activation differences in the PMC between the HOLD and CONTROL conditions even though these conditions certainly differ in WM load. Taken together, we cannot entirely exclude contributions of WM load to the activations observed in PMC. However, the discussed differences between the experimental conditions and the associated brain activity lead us to believe that this activation must at least in part be driven by the enhanced requirement for dynamic reactivation during rehearsal.

It could be speculated that the role of the PMC is related to covert motor plans. However, as the button‐press was performed only after the target stimulus presentation, and corresponding activity was regressed out by a response regressors of no interest, it is very unlikely that a preparation for the button‐press would relate to the observed premotor activity. It could further be speculated that PMC activity during the delay period relates to plans or minimal movements in the sense of active perception (Friston, Daunizeau, Kilner, & Kiebel, 2010; Gibson, 1966). The stimuli in our experiment were applied as passive touch, meaning that it was not necessary to press the finger on the Braille display to perceive the stimuli (because the display was taped to the fingertip). Nevertheless, most of human perception is active in nature, meaning that we typically explore our environment by performing motor actions that affect our sensory inputs. The mechanical stimulation of the fingertip might thus trigger minimal motor plans or movements, like the intent to hold the finger against the stimulation display. It is unlikely but conceivable that the mental replay thereof could lead to premotor activity reflecting motor planning. However, as we did not find motor cortex activity, and none of the participants reported motor movements during rehearsal, we consider it unlikely that premotor activity comes from micromovements.

Interestingly, in our previous tactile WM decoding studies we observed content‐specific codes in the PMC. Multivoxel activation patterns specific to particular WM items were found for both, vibration frequency (Schmidt et al., 2017) and patterned stimuli (Schmidt & Blankenburg, 2018), suggesting that the PMC might in fact be part of the WM storage network, despite showing a content‐independent activity. One interpretation might argue that the apparent content‐independence of PMC in the current study could be the result of abstraction (e.g., verbalization) of the rehearsed content und thus, would constitute a content storage across stimulus modalities. Although we cannot entirely rule out this possibility, the results of our DCM analysis suggest that the role of the PMC goes beyond mere content storage. The connectivity weights of the winning DCM indicate that PMC exerts a driving excitatory effect on IFG and SPL during rehearsal, which is well in line with the interpretation that the PMC activates a specific WM item representation within these content‐specific regions. Furthering this argument, it is important to consider that MVPA simply distinguishes between different activation patterns within a region and that the possibility to decode from a region does not automatically imply content storage. Instead, if a brain region implements a dynamic process that reactivates neuronal populations in content‐coding regions, it must generate distinct signals to specifically activate distinct neuronal populations that code for different contents. These different signals would in turn be amenable to MVPA. In light of these considerations, we must acknowledge that distinguishing content‐dependent from content‐specific brain regions based on multivoxel activation patterns is not a straight‐forward task, as long as we are ignorant to the nature of the representations driving the effects. Future studies using time‐resolved MVPA in combination with connectivity analyses may help elucidate this issue. These limitations considered, in our study, the right IFG and left SPL did activate for their preferred stimuli only, whereas the PMC activated for both pattern and frequency stimuli. This distinction is in line with previous reports and makes these regions excellent targets for future research into the neural substrates of WM storage and its dynamic components.

### 4.3 Rehearsal as an attentional mechanism

Our paradigm required participants to sequentially pull memorized contents into their focus of attention, that is, alter their focus of attention between different WM items. In classical WM studies, participants are required to retain only one item in WM for an extended period of time (like in the WM HOLD condition in the current study). In order to accomplish this task, participants may well refresh their WM representation sporadically in order to prevent the decay of activity in the corresponding content‐coding neuronal ensembles (which may explain the PMC activity found in these studies). However, the rate and timing at which such reactivations occur are uncontrolled and unknown making such paradigms unsuited to study the dynamic components of the WM system. In contrast, in our paradigm, participants sequentially prioritized different WM items, ensuring a continuous and timed update of their focus of attention. To test how this update is implemented in the dynamic interaction between the involved regions, we compared DCMs allowing for different inputs and connectivity modulations within the rehearsal network. The winning model featured direct input to the PMC, content‐specific excitatory connectivity modulation from PMC to SPL and IFG and content‐specific inhibitory connectivity modulation from SPL and IFG back to PMC, suggesting that PMC drives SPL and IFG during rehearsal. This finding aligns well with the idea that in order to update the focus of attention on a WM content, within the PMC an attentional signal is generated that refreshes activation in content‐coding regions. Such attentional signals are typically believed to rely on top‐down mechanisms (Katsuki & Constantinidis, 2014). However, given that there is no clear‐cut functional hierarchy between the investigated regions, our analysis cannot determine if the driving signal constitutes a top‐down, bottom‐up, or any alternative type of connection. Nevertheless, our results support a directed driving effect from the PMC onto SPL and IFG that underlies the sequential prioritizing of WM contents during rehearsal.

Different perspectives on the function of an attentional influence from the PMC are worth discussing. One perspective considers the role of the PMC in light of the “premotor theory of attention” (Rizzolatti & Craighero, 1998). Rizzolatti and colleagues argue that there is no need to consider attention and (intended) action as two independent processes, which rely on distinct anatomical circuits (Craighero, Fadiga, Rizzolatti, & Umiltà, 1999; Rizzolatti, Riggio, & Sheliga, 1994). Following this theory, the observed activity in the PMC could be associated with covert attention to the finger, realized by the same circuitry as an intended motor movement of the finger. As both of our experimental conditions require allocation of spatial attention to the finger, it appears plausible that our DCM results indicate that the PMC is driving the activity in SPL and IFG, as attention might be a prerequisite for rehearsal of a specific content.

Most experimental work on attentional mechanisms in WM stems from visual spatial attention and the preparation of eye movements, where the role of the frontal eye fields (FEFs), located in direct proximity to the PMC, is emphasized. Their common involvement in visual WM has been related to attentional selection mechanisms as they are part of rehearsal processes (Gazzaley & Nobre, 2012; Myers et al., 2017). In nonhuman primate studies, the interaction of PFC, FEFs, and posterior parietal cortex (PPC) has been investigated in electrophysiological studies and was found to be modulated by shifts of attention (Buschman & Miller, 2007) and WM (Salazar, Dotson, Bressler, & Gray, 2012). Likewise, in humans joint activation of PFC and PPC appear to support various cognitive processes and have been observed across WM tasks, attention tasks, forming intentions (e.g., planning of saccades) and others (Ikkai & Curtis, 2011), where the interaction of prefrontal and PPC in spatial visual WM tasks was related to the realization of priority maps of space (Jerde & Curtis, 2013; Jerde, Merriam, Riggall, Hedges, & Curtis, 2012). Interestingly, our conjunction analysis also revealed that parietal activity supports WM in general, although SPL activity was stronger for rehearsal of spatial information than frequency information. Further research is needed to dissect potential differences in the PFC and PPC interactions with regards to contributions of the PMC and the FEFs to attention allocation and the realization of representations of spatial information, for example, in the sense of representing spatial information as priority maps in a kind of body‐space representation.

### 4.4 Summary and outlook

Our results further support the dissociation of WM content representations of spatial layout and vibratory frequency in SPL and right IFG, respectively. In contrast, we found the PMC to activate during rehearsal independent of the rehearsed content, suggesting that it may be involved in implementing dynamic aspects of WM rather than content storage. Estimates of directed connectivity between these three regions by means of DCM analyses support the view that the PMC drives the activity in SPL and IFG during rehearsal and may implement an attentional signal that brings a specific WM item into the focus of attention, potentially by reactivating content‐coding neuronal ensembles as postulated by attention‐based refreshing.

Recently, Miller, Lundqvist, and Bastos (2018) emphasized the importance of beta‐gamma coupling for the interplay between content‐related activity and top‐down control mechanisms in WM. They presented data suggesting that deep‐layer beta oscillations mediate top‐down impact on gamma bursts in superficial layers, which are thought to code WM content. Interestingly, the retention of vibrotactile frequency has been demonstrated to induce parametrically modulated beta‐band oscillations in the right IFG (Spitzer et al., 2010). Similarly, content‐specific modulation of MEG gamma oscillations have been observed and localized to the right IFG (von Lautz et al., 2017). While these studies are compatible with beta‐gamma coupling for WM coordination, they do not allow dissociating reactivating top‐down processes from those reflecting content per se because the remembered content was always of the same format (i.e., frequencies). Accordingly, our findings warrant future M/EEG studies that utilize the rehearsal of vibrotactile frequency and spatial layout information to test the recent suggestions regarding beta‐gamma coupling, for example, by investigating if a source in the PMC drives the parametric beta‐band oscillations in the right IFG during rehearsal.

## CONFLICT OF INTEREST

The authors declare no competing financial interests.

## Data Availability

The data that support the findings of this study are available on request from the corresponding author. The data are not publicly available due to privacy or ethical restrictions.
